# Impacts of *perR* on oxygen sensitivity, gene expression, and murine infection in *Clostridioides difficile* 630∆*erm*

**DOI:** 10.1128/jb.00468-24

**Published:** 2025-01-23

**Authors:** Anna L. Gregory, Hailey E. Bussan, Madeline A. Topf, Andrew J. Hryckowian

**Affiliations:** 1 Department of Medicine, Division of Gastroenterology and Hepatology, University of Wisconsin School of Medicine and Public Health 5232 , Madison, Wisconsin, USA; 2 Department of Medical Microbiology & Immunology, University of Wisconsin School of Medicine and Public Health 5232 , Madison, Wisconsin, USA; 3 Microbiology Doctoral Training Program, University of Wisconsin-Madison 5228 , Madison, Wisconsin, USA; 4 Department of Bacteriology, University of Wisconsin-Madison 205263 , Madison, Wisconsin, USA; University of Illinois Chicago, Chicago, Illinois, USA

**Keywords:** *Clostridium difficile*, oxidative stress, anaerobes, transcriptional repression

## Abstract

*Clostridioides difficile* infection (CDI), characterized by colitis and diarrhea, afflicts approximately half a million people in the USA every year, burdening both individuals and the healthcare system. *C. difficile* 630Δ*erm* is an erythromycin-sensitive variant of the clinical isolate *C. difficile* 630 and is commonly used in the *C. difficile* research community due to its genetic tractability. 630Δ*erm* possesses a point mutation in *perR*, an autoregulated transcriptional repressor that regulates oxidative stress resistance genes. This point mutation results in a constitutively de-repressed PerR operon in 630Δ*erm*. To address the impacts of *perR* on phenotypes relevant for oxygen tolerance and relevant to a murine model of CDI, we corrected the point mutant to restore PerR function in 630∆*erm* (herein, 630∆*erm perR*^WT^). We demonstrate that there is no difference in growth between 630Δ*erm* and 630Δ*erm perR*^WT^ under anaerobic conditions or when exposed to concentrations of O_2_ that mimic those found near the surface of the colonic epithelium. However, 630∆*erm perR*^WT^ is more sensitive to ambient oxygen than 630∆*erm*, which coincides with alterations in expression of a variety of *perR*-dependent and *perR*-independent genes. Finally, we show that 630∆*erm* and 630∆*erm perR*^WT^ do not differ in their ability to infect and cause disease in a well-established murine model of CDI. Together, these data support the hypothesis that the *perR* mutation in 630∆*erm* arose as a result of exposure to ambient oxygen and that the *perR* mutation in 630∆*erm* is unlikely to impact CDI-relevant phenotypes in laboratory studies.

**IMPORTANCE:**

*Clostridioides difficile* is a diarrheal pathogen and a major public health concern. To improve humans’ understanding of *C. difficile*, a variety of *C. difficile* isolates are used in research, including *C. difficile* 630Δ*erm*. 630Δ*erm* is a derivative of the clinical isolate 630 and is commonly studied because it is genetically manipulable. Previous work showed that a mutation in *perR* in 630Δ*erm* results in a dysregulated oxidative stress response, but no work has been done to characterize *perR*-dependent effects on the transcriptome or to determine impacts of *perR* during infection. Here, we identify transcriptomic differences between 630∆*erm and* 630∆*erm perR*^WT^ exposed to ambient oxygen and demonstrate that there is no strain-based difference in burdens in murine *C. difficile* infection.

## INTRODUCTION

*Clostridioides difficile* is a leading cause of infectious diarrhea, resulting in an estimated 500,000 annual cases in the USA alone (1). A healthy microbiota typically prevents symptomatic *C. difficile* infection (CDI) through colonization resistance. However, ecological disturbances, commonly broad-spectrum antibiotics, disrupt the gut microbiota and give *C. difficile* access to vacant niches, which facilitates CDI (2–4). During dysbiosis, *C. difficile* can access nutrients otherwise consumed by the gut microbiota, overcome the effects of inhibitory metabolites produced by the microbiota/host, and increase in abundance in the gastrointestinal (GI) tract. During infection, *C. difficile* produces toxins (TcdA and TcdB; and CDT in hypervirulent strains), which induce host inflammation and are responsible for the diarrhea characteristic of CDI. This diarrhea contributes to transmission of *C. difficile* spores and allows *C. difficile* to gain a metabolic advantage by suppressing the recovery of the microbiota (3, 5–7). However, by inducing inflammation, *C. difficile* also causes the elevation of oxygen (O_2_) and reactive oxygen species (ROS) in the gut lumen (8–12). As an obligate anaerobe, *C. difficile* has evolved a variety of strategies to resist oxidative stress including sporulation, a versatile metabolism, and oxygen detoxification enzymes such as flavodiirons, rubrerythrins, and desulfoferrodoxin (Rbo) (9, 13–15). Previous work described various aspects of the response of *C. difficile* to oxidative stress (8, 16–18) and characterized proteins that play roles in oxidative stress tolerance (8–10, 16–19). One important regulator of a subset of these oxygen detoxification enzymes is the peroxide repressor (PerR).

PerR is an autoregulated transcriptional repressor and is a member of the ferric uptake repressor (Fur) family of proteins (19, 20). PerR is involved in oxidative stress responses in multiple bacterial species including *Clostridium acetobutylcium*, *Bacillus subtilis*, *Streptococcus pyogenes*, *Streptococcus mutans*, *Staphylococcus aureus*, *Campylobacter jejuni*, and *Clostridioides difficile* (21–27). Under anaerobic conditions, PerR binds to its target promoters and represses their expression. Oxidative stress triggers a metal-catalyzed histidine oxidation, and PerR undergoes a conformational change, causing it to release from its target promoters, which induces de-repression of the PerR regulon (20, 27). In *S. pyogenes,* a *perR* mutant was hyper-resistant to peroxide. However, it was highly attenuated in a murine model, demonstrating the importance of an appropriately regulated PerR regulon for virulence *in vivo* (22). A *perR* mutation identified in a *S. mutans* Δ*spxA1* strain similarly rendered PerR inactive, priming the strain to tolerate oxidative stress. However, this work also showed that PerR had a limited impact on the transcriptional response of *S. mutans* to hydrogen peroxide (24). In *C. acetobutylcium*, an obligate anaerobe, a *perR* mutant was more aerotolerant than wild type, and it was determined that PerR regulates oxidative stress genes, including reverse rubrerythrins, flavodiiron proteins (FDPs), and superoxide-reducing Dfx, as well as two putative enzymes involved in central energy metabolism (28, 29).

**Fig 1:**
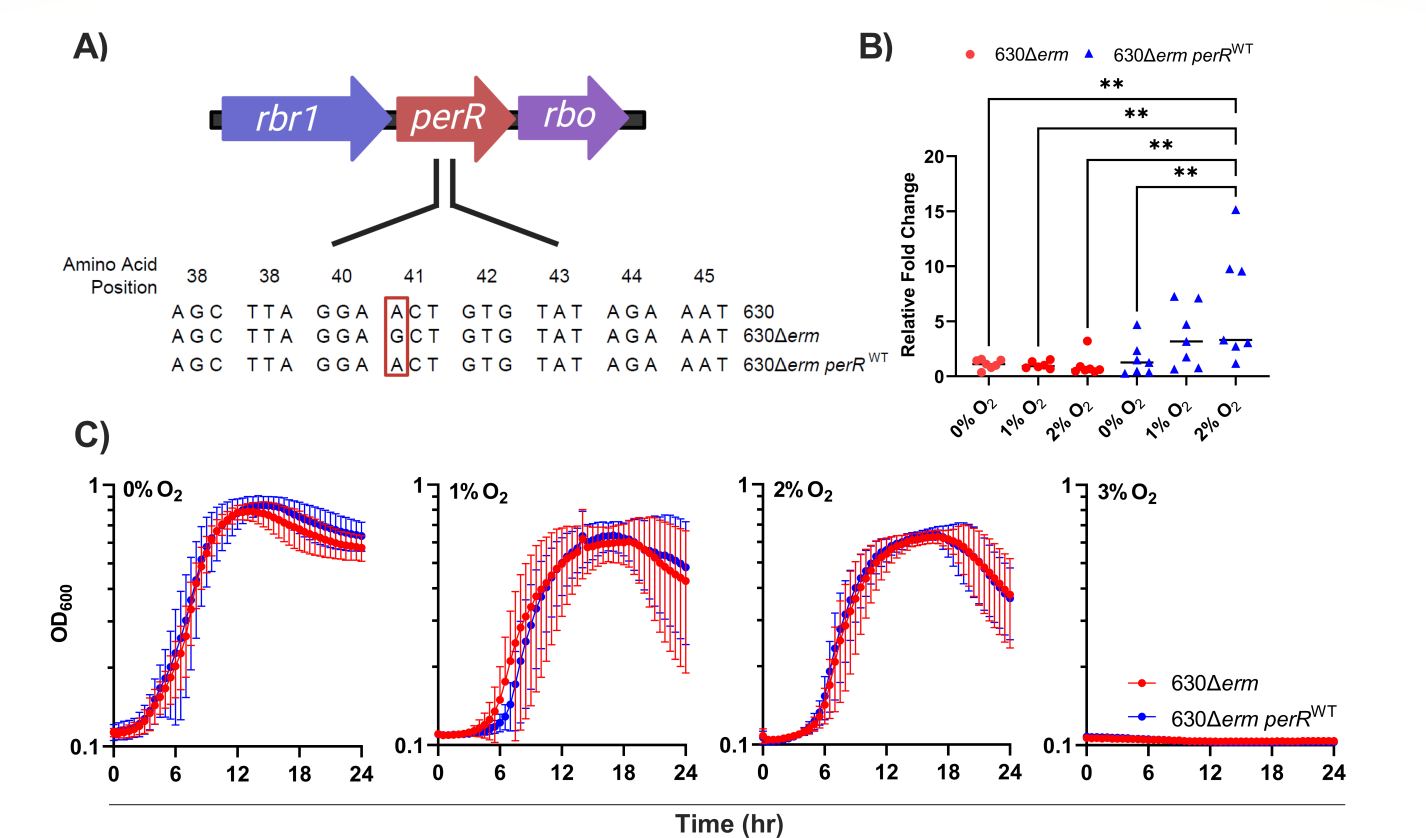
*In vitro* assays of 630Δ*erm* and 630Δ*erm perR*^WT^ exposed to physiologically relevant levels of O_2_. (**A**) Genomic context of *perR* in *C. difficile* 630, 630Δ*erm*, and 630Δ*erm perR*^WT^. The operon containing *perR* consists of three genes: a rubrerythrin (*rbr1*), the transcriptional repressor PerR (*perR*), and a desulfoferrodoxin (*rbo*). 630Δ*erm* has a point mutation in *perR*, resulting in a T41A amino acid substitution. (**B**) RT-qPCR of *perR* from 630Δ*erm* (red) and 630Δ*erm perR*^WT^ (blue) exposed to 0%, 1%, or 2% O_2_. Data points represent the relative fold change compared within each strain grown under anaerobic conditions, with *rpoC* used as the housekeeping gene (*n* = 7). Statistical significance was determined by two-way analysis of variance (ANOVA) with Tukey’s post-hoc test; **P < 0.01. (**C**) Growth curves of 630Δ*erm* (red) and 630Δ*erm perR*^WT^ (blue) in mRCM grown anaerobically or hypoxically in the presence of 1%, 2%, and 3% O_2_. Data points represent the mean OD_600_ (*n* = 6–8), and error bars represent the standard deviation. Panel A was created with Biorender.com under agreement #Z41E368.

In *C. difficile,* the operon containing *perR* consists of three genes: rubrerythrin (*rbr1*), *perR*, and a desulfoferrodoxin (*rbo*) (Fig. 1A). Genes within this operon are upregulated upon exposure to 1.5% O_2_ *in vitro* (8). In ex-germ-free mice mono-colonized with *C. difficile* and bi-colonized with *C. difficile* and *Bacteroides thetaiotaomicron*, the genes of the *perR* operon were among the 10% most abundant transcripts in *C. difficile* (2). Studies examining the activity of *rbo* demonstrated that when inactivated, *C. difficile* was more sensitive to oxygen exposure. Furthermore, when *C. difficile rbo* was expressed in *Escherichia coli*, it demonstrated superoxide scavenging activity (15). Taken together, these data indicate that *perR* expression is responsive to oxidative stress and that PerR and PerR-regulated genes are important for *C. difficile* to navigate oxidative stress both *in vitro* and perhaps *in vivo*.

*C. difficile* 630Δ*erm* is an erythromycin-sensitive, lab-generated derivative of *C. difficile* 630 (herein, 630∆*erm* and 630, respectively). 630∆*erm* is amenable to allelic exchange procedures and is therefore commonly used in the field for generating knockout mutants. Seven spontaneous mutations were previously identified in 630Δ*erm* relative to 630 (30). These mutations include a single nucleotide polymorphism (SNP) in *perR*, a SNP in *eutG*, a SNP in a transcriptional regulator of the GntR family (CD630_35630), and three SNPs in intergenic regions. Additionally, an 18 bp duplication is present in *spo0A* in 630Δ*erm* (30). The *perR* point mutation in 630Δ*erm* results in an amino acid substitution at position 41 (T41A) (Fig. 1A). This mutation affects the helix-turn-helix motif of the DNA-binding domain of PerR and results in a constitutively expressed *perR* operon regardless of O_2_ or H_2_O_2_ exposure. A constitutively expressed *perR* operon provides 630Δ*erm* with a higher tolerance to O_2_ and H_2_O_2_ than parental strain 630, and plasmid-based complementation of *perR* from 630 into 630∆*erm* restores H_2_O_2_-dependent expression of one PerR-repressed gene, *rbr1* (27).

Despite these previous findings on the role of *perR* in *C. difficile* and other organisms, there are several remaining questions relating to the direct effects of the *perR* point mutation in 630Δ*erm* on oxidative stress resistance, gene expression, and the resulting impacts on infection. These are important gaps in knowledge, as this point mutation may impact interpretation of previous and future data because 630Δ*erm* is such a widely used strain in the *C. difficile* field. To address these gaps, we corrected the *perR* point mutation in 630Δ*erm* to create 630∆*erm perR*^WT^. We demonstrate that this strain has a repressible *perR* operon and that there is no growth difference between 630Δ*erm* and 630∆*erm perR*^WT^ in the presence of 0%–3% O_2_. However, we show that 630Δ*erm* is fitter than 630∆*erm perR*^WT^ when exposed to ambient air (21% O_2_). We also characterize 630Δ*erm* and 630∆*erm perR*^WT^ transcriptomes exposed to ambient O_2_. Finally, using 630Δ*erm* and 630∆*erm perR*^WT^, we demonstrate that functional PerR does not impact *C. difficile* burdens or diarrhea in a murine model of CDI.

## RESULTS

### Targeted restoration of the mutant *perR* allele in 630∆*erm* and impacts on growth and survival in the presence of oxygen

To determine the impact of a constitutively expressed PerR on *in vitro* phenotypes, we corrected the point mutation in 630Δ*erm*, generating 630Δ*erm perR*^WT^ (Fig. 1A) (27). Correction of the point mutation was confirmed by whole-genome sequencing and alignment, comparing the parental 630Δ*erm* to 630∆*erm perR*^WT^. A single SNP at position 1,006,274 (G → A) was identified, indicating the only genetic difference between the two strains was the restoration of a wild-type *perR*.

To confirm wild-type PerR function in 630Δ*erm perR*^WT^, *perR*-specific RT-qPCR was performed on RNA extracted from 630Δ*erm* and 630Δ*erm perR*^WT^ grown in the presence of 0%, 1%, and 2% O_2_ (Fig. 1B). These O_2_ concentrations were selected because they reflect those present in the colon (31, 32). This analysis revealed that *perR* transcription was non-responsive to oxygen in 630Δ*erm*, due to constitutive de-repression of its operon. However, *perR* transcripts in 630Δ*erm perR*^WT^ were elevated as a function of increasing oxygen exposure, which demonstrates the *perR* operon is de-repressed upon exposure to 2% O_2_. These data confirm that correcting the *perR* point mutation restored a wild-type, oxygen-responsive phenotype and are supported by previously published RNA-seq data that showed that *perR* is upregulated in 630 at 1.5% O_2_ (8).

To evaluate the impacts of oxygen-responsive *perR* expression on *C. difficile* growth, 630Δ*erm* and 630Δ*erm perR*^WT^ were grown in a complex, rich medium (modified Reinforced Clostridial Medium [mRCM]) in the presence of 0%, 1%, 2%, and 3% O_2_ (Fig. 1C; Fig. S1) (3, 33). We did not observe differences in growth kinetics between the two strains at these O_2_ concentrations. However, the growth of both strains was negatively impacted by increasing O_2_ concentration, and no growth was observed in 3% O_2_. These data partially replicate results of a recent study on O_2_ reductases in *C. difficile*. There, targeted restoration of PerR function in 630∆*erm* did not impact *C. difficile* growth at 1% O_2_ (34).

**Fig 2:**
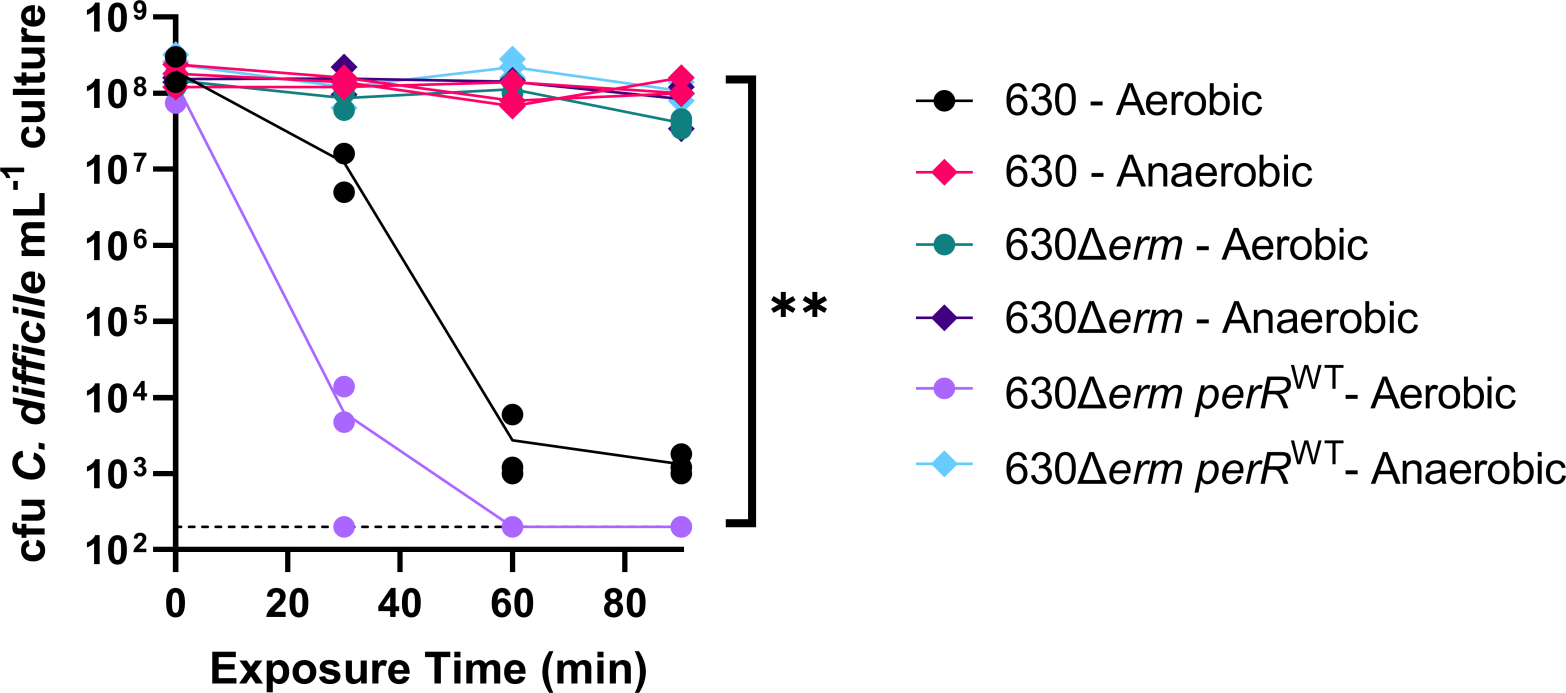
Aerotolerance assay of 630, 630Δ*erm*, and 630Δ*erm perR*^WT^ exposed to ambient air. Aliquots of stationary phase cultures of each strain were exposed to ambient air for 0, 30, 60, and 90 minutes, or maintained under anaerobic conditions and plated on pre-reduced CDMN agar at the indicated timepoints. Colonies were quantified after overnight growth in an anaerobic chamber (*n* = 3 cultures per strain per condition). Statistical testing was determined by two-way analysis of variance (ANOVA); **P < 0.01.

Despite differences in PerR activity between 630∆*erm* and 630∆*erm perR*^WT^, a constitutively de-repressed PerR regulon offers no apparent fitness advantage at physiologically relevant O_2_ concentrations (Fig. 1). Previous work showed that 630∆*erm* has increased survival relative to 630 upon exposure to ambient O_2_ (27). To determine if 630∆*erm perR*^WT^ has restored sensitivity to an ambient air (~21% O_2_), an ambient air exposure assay was performed. Cell viability of 630, 630Δ*erm*, and 630Δ*erm perR*^WT^ was quantified after exposure to ambient air for 0 to 90 minutes. Viability of 630 and 630Δ*erm perR*^WT^ were decreased upon exposure to ambient air. However, this exposure did not have an impact on 630∆*erm,* demonstrating that targeted restoration of *perR* restores wild-type levels of sensitivity to ambient air in *C. difficile* (Fig. 2).

### Differences in 630Δ*erm* and 630Δ*erm perR*^WT^ transcriptomes as a function of ambient air exposure

**Fig 3:**
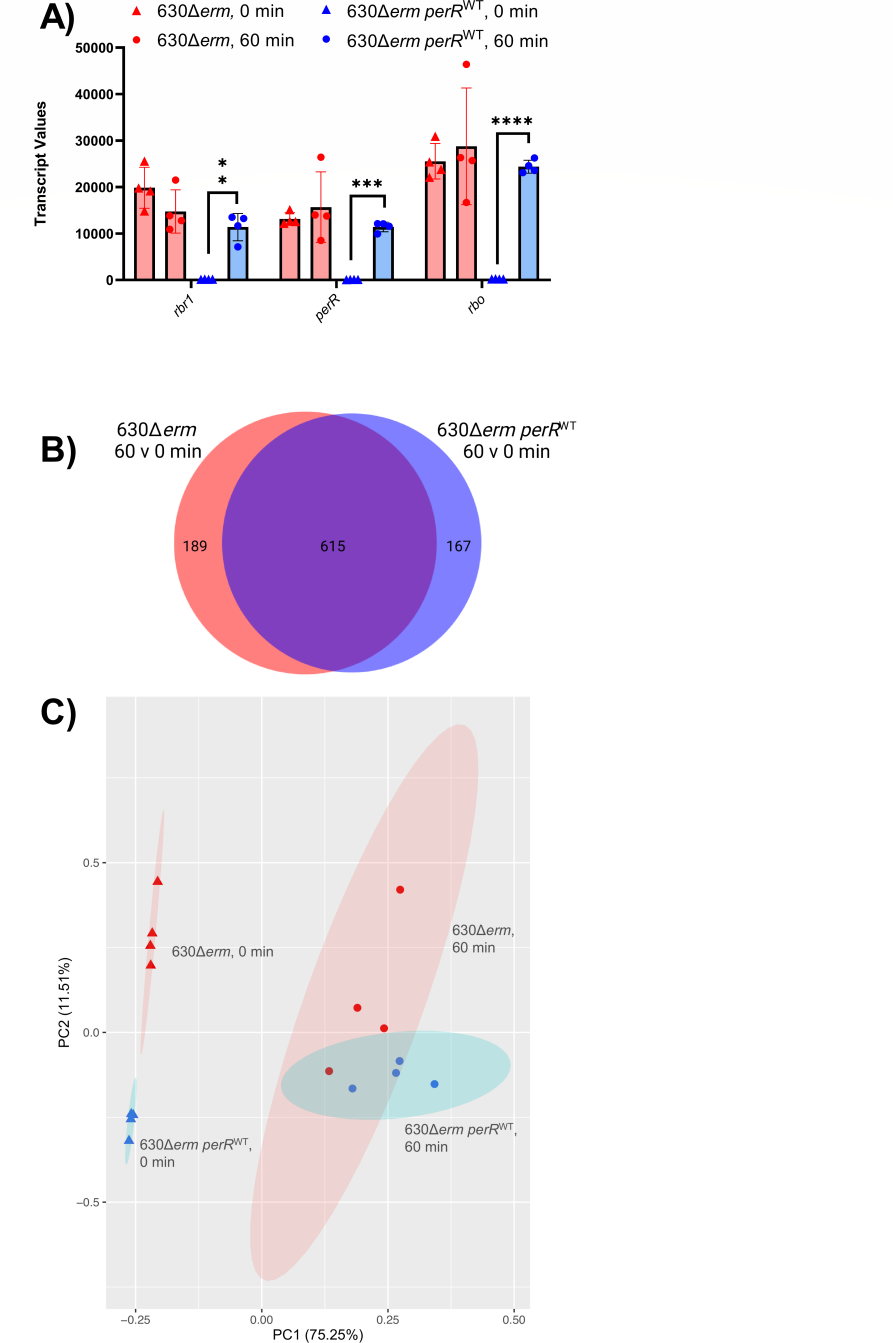
Transcriptional profiling of 630Δ*erm* and 630Δ*erm perR*^WT^ exposed to ambient air. 630Δ*erm* and 630Δ*erm perR*^WT^ were grown to mid-log phase in mRCM and then exposed to ambient air for 0 and 60 minutes. RNA-seq was performed on *n* = 4 independent cultures, for each strain at each timepoint. (**A**) Transcript values of the genes in the *perR* operon (*n* = 4). Statistical testing was determined by paired *t*-test; *P < 0.05, **P < 0.01, ***P < 0.001, and ****P < 0.0001. (**B**) A Venn diagram illustrating that the majority of significantly differentially regulated genes (FC ≥ |2|, *P* < 0.05) overlapped between the 630Δ*erm* and 630Δ*erm perR*^WT^. (**C**) Principal component analysis (PCA) plot of RNA-seq data from 630Δ*erm* (red) and 630Δ*erm perR*^WT^ (blue) exposed to ambient air for 0 (triangle) and 60 (circle) minutes. Ellipses represent 95% confidence intervals based on strain and time of ambient air exposure. Panel B was created with Biorender.com under agreement #N27K433.

To better understand genes involved in 630∆*erm* resistance to ambient air (Fig. 2), we performed RNA-seq on 630Δ*erm* and 630Δ*erm perR*^WT^ at 0 and 60 minutes post-ambient air exposure and compared transcriptomes between the two strains and two timepoints (Tables S2 to S5). The 60 minute timepoint was chosen based on RT-qPCR of *perR* transcripts from total RNA extracted from 630 at 0, 15, 30, and 60 minutes post-air exposure (Fig. S2), which showed elevated *perR* transcripts at both 30 and 60 minutes of aerobic exposure compared to anaerobic control. RNA extracted at 60 minutes was high quality via Bioanalyzer (data not shown) and therefore selected as the timepoint. RNA-seq showed that the operon containing *perR* was responsive to aerobic exposure in 630Δ*erm perR*^WT^ but was expressed at high levels regardless of aerobic exposure in 630∆*erm* (Fig. 3A). In addition to the genes in this operon, transcripts of five other genes were more abundant under anaerobic conditions in 630∆*erm* relative to 630∆*erm perR*^WT^ (Table 1), suggesting a small subset of genes are repressed by PerR in *C. difficile*. Similarly, there were largely overlapping patterns of gene expression for both strains between 0 and 60 minutes of ambient air exposure. Specifically, although there were differences in cell viability at the 60 minute timepoint (Fig. 2), both strains shared 615 differentially regulated genes (Fig. 3B), many of which were previously predicted/characterized to be involved in oxidative stress resistance (Table S6). This similarity in transcriptional response to ambient air is also evident from principal component analysis (Fig. 3C), which shows that both strain and exposure to O_2_ had a significant impact on the transcriptome (the impact of O_2_ exposure was more significant), but the interaction of the variables was not significant (permutation multivariate analysis of variance [PERMANOVA] ~ time * genotype, time *P* = 0.001; strain *P* = 0.006, time * strain, *P* = 0.085).

**TABLE 1:** Significantly upregulated genes in 630Δ*erm* relative to 630Δ*erm perR*^WT^ under anaerobic conditions^*a*^

Locustag	Gene	Description	LogFC^*b*^	*P* value
CDIF630erm_00875		Putative diguanylate kinase signaling protein	2.415049	0.003524
CDIF630erm_00944	*rbr1*	Putative rubrerythrin	6.815261	7.17E-14
CDIF630erm_00945	*perR*	Peroxide-responsive repressor	6.884237	7.34E-14
CDIF630erm_00946	*dsr (rbo*)	Desulfoferrodoxin	6.840431	3.36E-14
CDIF630erm_00947		Oxidative stress glutamate dehydrogenase (glutamate synthase-like) (rubredoxin)	6.307636	1.02E-13
CDIF630erm_00948		Putative metallo-beta-lactamase superfamily protein	2.727654	6.00E-09
CDIF630erm_01262		Putative conjugative transposon protein	2.715317	0.000238
CDIF630erm_01792		Putative diguanylate kinase signaling protein	2.082062	0.00484

^*a*^ RNA from midlogarithmic phase cultures was extracted before exposure to ambient oxygen, RNA-seq was performed, and data were analyzed as described in the Materials and Methods.

^*b*^ LogFC, Log fold change.

These data suggest that the increased viability of 630∆*erm* relative to 630Δ*erm perR*^WT^ could be due to elevated levels of PerR-dependent gene products present in 630∆erm cells prior to oxygen exposure, which would prime the cells for oxidative stress. Beyond *rbr1*, *perR*, and *rbo*, five genes were upregulated in 630∆*erm* relative to 630Δ*erm perR*^WT^ at the *t* = 0 timepoint Table 1. These genes include a putative oxidative stress glutamate dehydrogenase (CDIF630erm_00947), a putative metallo-beta-lactamase superfamily protein (CDIF630erm_00948), a putative conjugative transposon protein (CDIF630erm_01262), and two putative diguanylate kinase signaling proteins (CDIF630erm_00875 and CDIF630erm_01792). These data suggest that although PerR impacts *C. difficile* survival in ambient air, PerR does not exert major control, outside of the *perR* operon, over oxidative stress resistance genes in *C. difficile*, as previously observed in other microbes (21, 22, 24, 25, 28, 29).

### 630Δ*erm* and 630Δ*erm perR*^WT^ do not differ in their ability to infect or cause diarrhea in mice

**Fig 4:**
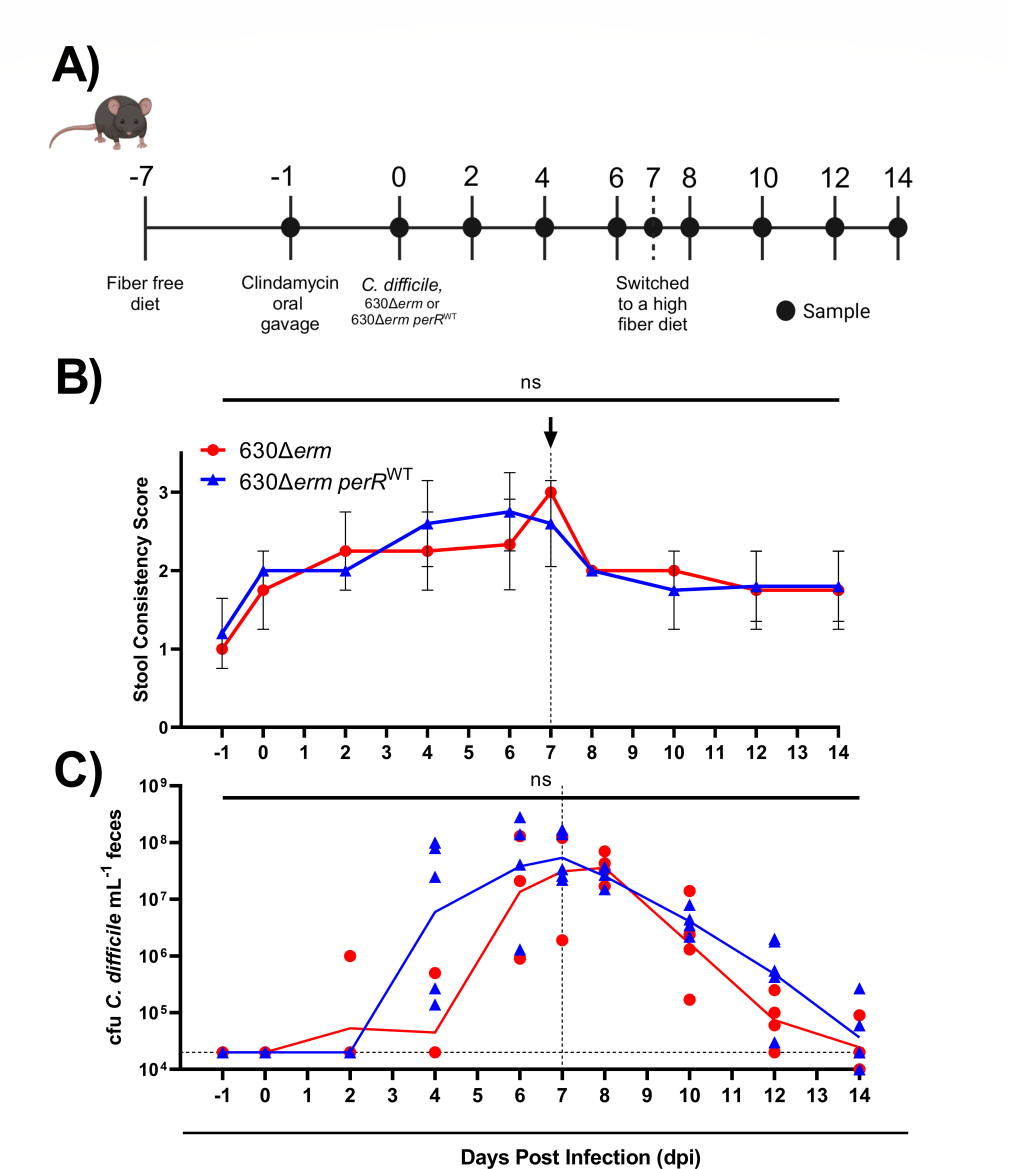
CDI in mice infected with 630Δ*erm* and 630Δ*erm perR*^WT^. (**A**) Conventional, age-matched male C57BL/6 mice were placed on a fiber-free diet, gavaged with clindamycin, and infected with either 630Δ*erm* or 630*Δerm perR*^WT^ (*n* = 4–5). Mice were switched to a high-fiber diet 7 days after being infected with *C. difficile*. (**B**) Average stool consistency scores: 1 = hard, dry pellet; 2 = soft but fully formed pellet; 3 = runny, poorly formed pellet. (**C**) The geometric means of *C. difficile* burdens for each strain over the experimental time course. The limit of detection for this assay is indicated in the horizontal dashed line at 20,000 CFU *C*. *difficile*/mL feces. Statistical testing was performed on CFU counts and stool consistency from mice between strains on each day and was not significant (ns) via Mann-Whitney test. Panel A was created with Biorender.com under agreement #Z41E368. The vertical dashed lines at 7 days post-infection in panels B and C indicate when the mice were switched from a fiber-free to a high-fiber diet.

During infection, *C. difficile* toxins induce host inflammation and elevate ROS (35–37). Therefore, given that 630Δ*erm* and 630Δ*erm perR*^WT^ differ in their ability to survive oxidative stress, we sought to determine the impact of *perR* on strain fitness during infection. To examine this, we leveraged a well-established murine model of CDI (33, 38). Mice were placed on a fiber-free (FF) diet and gavaged with clindamycin as in Figure 4A to reduce colonization resistance against *C. difficile*. Then, mice were gavaged with either 630Δ*erm* or 630Δ*erm perR*^WT^ to establish CDI. After 1 week of infection, the mice were switched to a high-fiber diet to determine if fiber-dependent CDI clearance kinetics differ between the strains (38).

Consistent with previous literature using 630 in murine models (39, 40), both 630∆*erm* and 630∆*erm perR*^WT^ induced mild CDI symptoms. Specifically, there was no difference between the two strains in stool consistency scores nor *C. difficile* burdens across the 14 days (Fig. 4B and C). All mice experienced an increase in stool softness on 0 day post-infection (dpi) due to administration of clindamycin (Fig. 4B). However, after infection with *C. difficile,* mice experienced an increase in diarrhea, regardless of the strain with which they were infected. For both strains, fecal burdens of *C. difficile* were similar when on the fiber-free diet and decreased once the mice were placed on the high-fiber diet, as previously demonstrated (Fig. 4C) (38). Interestingly, there was a slight delay in *C. difficile* burdens in both strains at early timepoints. Specifically, consistent burdens were not seen until 4 dpi, possibly due to sensitivity of the strains to clindamycin (Fig. S3). Taken together, these data demonstrate that 630Δ*erm* and 630Δ*erm perR*^WT^ do not differ in their ability to infect or cause diarrhea in mice.

## DISCUSSION

630Δ*erm* has been an important strain for elucidating the physiology and pathogenesis of *C. difficile* because of its extensive use to generate mutants via allelic exchange procedures. However, there are several mutations in 630∆*erm* relative to its parent strain (a clinical isolate) that may impact the generalizability of the findings made in 630∆*erm* to other *C. difficile* strains. The mutations in 630∆*erm* include a point mutation in *perR* that renders its PerR regulon constitutively de-repressed. Despite knowledge of this *perR* point mutation and its impacts on expression of genes within the *perR* operon, the effects of this mutation on *C. difficile* oxidative stress resistance, on the broader *C. difficile* transcriptome, and on CDI phenotypes remained poorly characterized.

Here, we restored a wild-type copy of *perR* in 630∆*erm* to create 630Δ*erm perR^WT^*. We determined that there is no difference in growth between 630Δ*erm* and *630*Δ*erm perR^WT^* at physiologically relevant O_2_ levels (Fig. 1). However, using these strains, we showed that a constitutively de-repressed PerR regulon allows *C. difficile* 630Δ*erm* to tolerate ambient air exposure (Fig. 2). Previous work that compared the survival of *C. difficile* exposed to ambient O_2_ compared 630 and 630∆*erm,* which have multiple genetic differences in addition to the *perR* point mutation (27). Our results support the previous findings and the hypothesis that the differences in oxidative stress resistance in these strains were due to *perR* and not to these other mutations. In addition, given that the T41A point mutation in *perR* is unique to 630Δ*erm* when compared to 11 diverse clinical isolates of *C. difficile* (including its parent strain 630) (27), it is reasonable to assume that this mutation was selected due to oxygen exposure during laboratory passage.

To better understand the genes repressed by PerR (which likely contribute to increased O_2_ tolerance by 630∆*erm*), we performed RNA-seq on 630∆*erm* and 630∆*erm perR*^WT^ exposed to ambient air (Fig. 3). This analysis suggested that PerR represses a small fraction of genes in *C. difficile*. Under anaerobic conditions, eight genes were upregulated in 630∆*erm* relative to 630∆*erm perR*^WT^ (Table 1; Table S2). This includes the genes present in the *perR* operon (*rbr1*, *perR*, and *rbo*), a putative oxidative stress glutamate dehydrogenase, a putative metallo-beta-lactamase, a putative conjugative transposon protein, and two putative diguanylate kinase signaling proteins. Our RNA-seq data also show that large-scale changes to the *C. difficile* transcriptome occur at 60 minutes post-air exposure, regardless of whether the strain has functional PerR. Specifically, 615 shared genes are differentially regulated in both strains at 60 minutes post-exposure to ambient air relative to the pre-exposure timepoint. This include genes involved in oxidative stress resistance (Table S6), many of which are likely under the control of other oxidative stress regulators (e.g., σ^B^) (12–14, 34, 41). Despite similarities of the transcriptional responses of 630∆*erm* and 630∆*erm perR*^WT^ to ambient air, 630∆*erm* and 630∆*erm perR*^WT^ have some differences in their responses to this treatment (Fig. 3). Deeper analysis of functional categories of these genes (Tables S6 to S8) revealed oxygen-dependent upregulation of genes involved in Stickland fermentation, ribosome synthesis, and the CRISPR system in 630∆*erm* and oxygen-dependent downregulation of peptidoglycan and teichoic acid metabolism in 630∆*erm perR*^WT^. These changes in gene expression mirror the differential survival of these two strains in the presence of oxygen (42).

Our data also indicate that there is no difference between 630Δ*erm* and *630*Δ*erm perR^WT^* in *C. difficile* burdens nor severity of infection in a murine model of CDI (Fig. 4). While our work was performed in conventional mice, previous work showed that *rbo*, *perR*, and *rbr* were among the top 10% most highly expressed genes in gnotobiotic mice infected with 630 (2), suggesting that PerR-dependent gene expression is important during infection. Because longitudinal and radial oxygen gradients are present in the gastrointestinal tract and O_2_ levels can be elevated by antibiotic treatment, it is possible that *C. difficile* encounters enough O_2_ to de-repress PerR-dependent genes during the onset, establishment, or maintenance of murine infection (31, 32, 41, 43). Therefore, the mouse experiments performed may disguise possible differences in fitness (positive or negative) due to a constitutively de-repressed PerR operon.

In summary, our work establishes that a constitutively de-repressed PerR regulon offers no fitness advantage at O_2_ levels encountered in the distal GI tract (Fig. 1) nor in a mouse model of CDI (Fig. 4). However, a constitutively de-repressed PerR regulon provides *C. difficile* with tolerance to ambient air (Fig. 2) and impacts gene expression in *C. difficile* in the presence and absence of oxygen (Fig. 3). Unique to 630Δ*erm*, the *perR* mutation invokes consideration of selective pressures that this strain may have encountered during exposure to ambient air in laboratory settings (27). This study adds to a growing body of literature on the ways in which obligate anaerobes resist oxidative stress and will contribute to future work in understanding these responses in *C. difficile*.

## MATERIALS AND METHODS

### Bacterial strains and culture conditions

*C. difficile* strains 630, 630Δ*erm*, and 630Δ*erm perR*^WT^ (44, 45) were maintained as −80°C stocks in 25% glycerol under anaerobic conditions in septum-topped vials. *C. difficile* strains were struck out on *Clostridioides difficile* agar with moxalactam and norfloxacin (CDMN agar), which is *C. difficile* agar base (Oxoid) supplemented with 32 mg/L moxalactam (Santa Cruz), 12 mg/L norfloxacin (Sigma-Aldrich), and 7% defibrinated horse blood (HemoStat Laboratories), and were cultured anaerobically for 24 hours. A single colony was picked into 5 mL of pre-reduced BD Difco reinforced clostridial medium (RCM) or a modified RCM (mRCM; RCM without soluble starch and agar) (33). Liquid cultures were grown at 37°C anaerobically for 16–24 hours and used as inocula for growth curves, aerotolerance assays, RNA-seq, RT-qPCR, and murine experiments. All bacterial growth media were pre-reduced for at least 24 hours in an anaerobic chamber (Coy) prior to use in experiments.

For *in vitro* growth curves, subcultures were prepared at 1:100 dilution in mRCM (3). Growth curves were performed anaerobically or in a hypoxic chamber at 1%, 2%, and 3% O_2_ (Coy). Clindamycin sensitivity growth curves were performed anaerobically in mRCM with clindamycin concentrations spiked into each well. All growth curves were performed in sterile polystyrene 96-well tissue culture plates (Falcon) with low-evaporation lids using a BioTek Epoch2 plate reader at 30 minute intervals. Plates were shaken on the orbital setting for 10 seconds before each read. The OD_600_ of the cultures was recorded using Gen5 software (version 3.10.06).

### Generation of *C. difficile* 630Δ*erm perR*^WT^

The *perR* point mutation was corrected using the PyrE allelic exchange system (46). Primers indicated in Table S1 were used to amplify *perR* from 630 genomic DNA. The amplicon containing *perR* from 630 was ligated into pMTL-YN3 after AscI and SbfI digestion of the vector and was inserted using New England Biolabs Quick Ligation Kit (M2200S). The plasmid construct was transformed and propagated into One Shot Top10 *E. coli* (Invitrogen) before transformation into conjugation-proficient *E. coli* HB101/pRK24 cells. The pMTL-YN3 with 630 *perR* was conjugated into 630∆*erm*∆*pyrE*. Plasmid integrants were selected using brain-heart infusion supplemented (BHIS) agar containing thiamphenicol (10 or 15 µg/mL), cefoxitin (8 µg/mL), kanamycin (30 or 50 µg/mL), and uracil (5 µg/mL). Double crossover events were selected using a defined minimal medium for *C. difficile* (CDDM) supplemented with uracil (5 µg/mL) and 5-fluoroorotic acid (2 mg/mL). Whole colony PCR using GoTaq Green Master Mix (Promega) amplified *perR* from potential clones. HypC4III digestion of PCR-purified *perR* (Zymo DNA Clean and Concentrator-5) confirmed 630 *perR* integration as this restriction enzyme digests 630 *perR* but is unable to recognize that cut site in 630Δ*erm* due to the point mutation (Fig. S4). After confirmation of 630 *perR* integration, the *pyrE* locus was restored using pMTL-YN1C to generate 630Δ*erm perR*^WT^.

Illumina whole-genome sequencing on DNA extracted (47) from 630Δ*erm* and 630Δ*erm perR*^WT^ was performed by Microbial Genome Sequencing Center (MiGS) with a minimum read count of 1.33 million reads (200 Mbp) per sample. Raw sequencing data were assembled using a reference-guided assembly pipeline (https://github.com/pepperell-lab/RGAPepPipe_CHTC) to *C. difficile* strain 630Δ*erm* (GenBank: LN614756.1) as previously described (48). Briefly, Fastqc v0.12.1 (49) assessed the quality of the sequences, which were then trimmed using Trimmomatic v0.39 (50). Sequences were aligned to the reference using BWA Mem v0.7.18 (51), and alignments were processed using SAMtools v1.21 (52). Picard v 2.18.25 (https://github.com/broadinstitute/picard) was used to remove duplicates and add read groups. Pilon v 1.78 (53) identified variants. Assembly and alignment quality was determined using Qualimap BamQC v2.2.1 (54). The mean coverage was 160× for 630Δ*erm* and 50× for 630Δ*erm perR*^WT^. Both samples had >95% aligned reads to the reference. To identify the SNP difference between 630Δ*erm* and 630Δ*erm perR*^WT^, a variant call format (VCF) was created using SnpSites v2.5.1 (55).

### Aerotolerance assays

Liquid cultures of 630, 630Δ*erm*, and 630Δ*erm perR*^WT^ were grown anaerobically in RCM overnight. Each culture (200 µL) was aliquoted into sterile polystyrene 96-well tissue culture plates, with four replicate plates set up, and was exposed to ambient air for 0, 30, 60, and 90 minutes at room temperature. Aeration of cultures using a multichannel pipette was performed immediately after removing from the chamber and approximately every 10 minutes throughout the assay. At each timepoint, one of the 96-well plates was passaged back into the anaerobic chamber. Serial dilutions were performed using pre-reduced phosphate-buffered saline (PBS) and plated on pre-reduced CDMN. Plates were incubated anaerobically for 24 hours at 37°C, and colonies present on CDMN plates were quantified.

### Transcriptional profiling of *C. difficile* response to ambient air

*C. difficile* 630Δ*erm* and 630Δerm *perR*^WT^ overnight cultures were back-diluted 1:100 into 35 mL of pre-reduced mRCM in Erlenmeyer flasks and were incubated anaerobically at 37°C until cultures reached a mid-log phase (OD_600_ = 0.3–0.4). At mid-log phase, 5 mL aliquots of the cultures were diluted 1:1 in chilled 1:1 ethanol:acetone and were stored at −20°C to preserve RNA. The remaining cultures were aerobically shaken (220 rpm) at 37°C for 60 minutes. After 60 minutes, 5 mL aliquots were diluted 1:1 in chilled 1:1 ethanol:acetone and were stored at −20°C to preserve RNA (56).

RNA was extracted by centrifuging samples at 3,000 × *g* for 5 minutes at 4°C. Pellets were washed with 5 mL cold, nuclease-free PBS and were centrifuged at 3,000 × *g* for 5 minutes at 4°C. The supernatant was removed, and remaining pellets were resuspended in 1 mL TRIzol and processed using a TRIzol Plus RNA Purification Kit (Thermo) with on-column DNase treatment. Purified RNA integrity was confirmed via 2100 Agilent BioAnalyzer and was frozen at −80°C.

RNA-seq was performed by MiGS on high-quality rRNA-depleted RNA extracts (12 million paired-end reads per sample). Quality control and adapter trimming were performed with bcl2fastq (version 2.20.0.445) (57). Read mapping was performed with HISAT2 (version 2.2.0) (58). Read quantification was performed using Subread’s featureCounts (version 2.0.1) (59) functionality. Read counts were loaded into R (version 4.0.2) (60) and were normalized using edgeR’s (61) Trimmed Mean of M values (TMM) algorithm (version 1.14.5). Subsequent values were then converted to counts per million (cpm). Differential expression analysis was performed using edgeR’s exact test for differences between two groups of negative-binomial counts with an estimated dispersion value of 0.1. Transcript level quantification, count normalization, and differential expression analysis were provided using *C. difficile* 630Δ*erm* (GCA_002080065.1_ASM208006v1) as the reference genome.

The principal component analysis (PCA) plot was generated from the RNA-seq data in R using version 4.4.1. PCA was performed using *prcomp* from *stats* package (version 4.4.1) and was visualized using *ggplot2* (version 3.5.1). Confidence ellipses were generated using *stat_ellipse* as implemented in *ggplot2*. PERMANOVA was assessed by *vegan::adonis2* (version 2.6-6.1).

### RT-qPCR of *perR* in *C. difficile* exposed to varying levels of O_2_

To identify a timepoint for RNA-seq at which *perR* was de-repressed, *C. difficile* 630 overnight cultures were back-diluted 1:100 into 35 mL of pre-reduced RCM and were incubated at 37°C until it reached a mid-log phase (OD_600_ = 0.3–0.4). At mid-log phase, one set of cultures was incubated aerobically at 37°C, shaking at 220 rpm, while the other culture was incubated at 37°C anaerobically. After 0, 15, 30, and 60 minutes, 5 mL aliquots of the cultures were diluted 1:1 in chilled 1:1 ethanol:acetone and were stored at −20°C to preserve RNA. RNA was extracted as described above.

RT-qPCR was performed using GoTaq 1-Step RT-qPCR Master Mix (Promega) according to the manufacturer’s instructions, with a concentration of 1 ng/µL RNA in a final volume of 10 µL. Each reaction was run with three technical replicates. The RT-qPCRs were performed on a QuantStudio 7 Flex (Applied Biosystems), and the threshold cycle (Ct) was determined using QuantStudio Real-Time PCR Software v1.7.2. The cycle run involved a reverse transcriptase activation and inactivation step of 40°C for 15 minutes and 95°C for 10 minutes. The PCR cycle was 95°C for 10 seconds, 60°C for 30 seconds, and 72°C for 30 seconds for 40 cycles, with a melt curve performed afterward. Relative fold change was determined by comparing the Ct values to the average Ct value at 0 minutes for each condition.

To quantify *perR* transcript levels of *C. difficile* 630Δ*erm* and *C. difficile* 630Δ*erm perR*^WT^ exposed to low levels of O_2_, overnight cultures of each strain were back diluted 1:50 into 14 mL of pre-reduced mRCM in 100 × 15 mm circular petri dishes (VWR) to optimize exposure to oxygen. Cultures were incubated at 37°C in 0%, 1%, and 2% O_2_ until they reached a mid-log phase (OD_600_ = 0.3–0.4). At mid-log phase, 5 mL aliquots of the cultures were diluted 1:1 in chilled 1:1 ethanol:acetone and were stored at −20°C to preserve RNA, and RNA was extracted as described above. RT-qPCR was performed using GoTaq 1-Step RT-qPCR Master Mix (Promega), with a concentration of 2 ng/µL of RNA in a final volume of 20 µL. Each reaction was set up with primers amplifying *perR* or *rpoC* (housekeeping gene) (Table S1), with two to three technical replicates. The RT-qPCRs were performed on a QuantStudio 7 Flex (Applied Biosystems) as previously described. Relative fold change was determined using the 2^-ΔΔCT^ method by comparing the Ct values to the anaerobic Ct value within each strain (62).

### Murine model of *C. difficile* infection (CDI)

All animal studies were carried out in strict accordance with the University of Wisconsin-Madison Institutional Animal Care and Use Committee (IACUC) guidelines (Protocol #M006305). CDI murine model was performed on age- and sex-matched, conventionally reared C57BL/6 mice bred in-house. Mice used in experiments were between 6 and 9 weeks of age. Mice were fed a fiber-free (FF) diet (Inotiv TD.150689) 1 week before antibiotic exposure. Mice were given a single dose of clindamycin by oral gavage (1 mg/mouse; 200 µL of 5 mg/mL solution); 24 hours later, they were given 200 µL of 630Δ*erm* or 630Δ*erm perR*^WT^ overnight cultures grown in RCM, via oral gavage (*n* = 4–5 mice per condition; average inoculum 8.6 × 10^7^ CFU/mL).

At 7 dpi, mice were switched from the FF diet to a fiber-rich standard rodent chow (Inotiv Teklad 2916) to observe fiber-dependent *C. difficile* clearance kinetics (33, 38). Throughout the entire experiment, feces were collected from mice directly into a microcentrifuge tube and were kept on ice. To quantify *C. difficile* burdens, 1 µL of each fecal sample was collected with a disposable inoculating loop and was resuspended in 200 µL PBS. Tenfold serial dilutions of fecal suspension were prepared in sterile polystyrene 96-well tissue culture plates (Falcon). For each sample, 10 µL aliquots of each dilution, with two technical replicates, were spread on CDMN agar. CDMN plates were incubated anaerobically at 37°C for 16–24 hours. Colonies were quantified and technical replicates were averaged to determine *C. difficile* burdens (limit of detection = 2 × 10^4^ CFU/mL). Stool consistency scores were also noted while processing fecal pellets. Fecal pellets were assigned a score of 1 = hard, dry pellets, difficult to transect with a disposable plastic culture loop; 2 = soft, fully formed pellets, easy to transect with a culture loop; or 3 = runny, poorly formed pellets, no pressure required to transect with a culture loop (3).

### Statistical analysis

All statistical analyses, except for the PERMANOVA, were performed using GraphPad Prism 9.4.1. PERMANOVA was performed as described in “PCA Generation and RNA-seq Data Analysis” section. Details of specific statistical analyses are indicated in figure legends. For all figures, *P < 0.05, **P < 0.01, ***P < 0.001, and ****P < 0.0001.

## Data Availability

Data on normalized transcript abundance and differential expression analysis are found in Tables S2 to S5. The raw data from the RNA-seq experiments shown in Figure 3 and Tables S2 to S5 and the raw data from whole genome sequencing of 630∆*erm* and 630∆*erm perR*^WT^ are available on NCBI Gene Expression Omnibus [GSE280615] and NCBI Sequence Read Archive [PRJNA1185794], respectively.

## SUPPLEMENTAL MATERIAL

Figure S1 jb.00468-24-s0001.pdf Growth curves of (A) 630Δ*erm* and (B) 630Δ*erm perR*^WT^ grown in mRCM at 0, 1, 2, and 3% O_2_.

Figure S2 jb.00468-24-s0002.pdf RT-qPCR of *perR* transcripts from 630.

Figure S3 jb.00468-24-s0003.pdf Growth curves of 630, 630Δ*erm*, and 630Δ*erm perR*^WT^ in the presence of clindamycin.

Figure S4 jb.00468-24-s0004.pdf HypC4III digest of *perR*-containing PCR amplicons.

Supplemental tables jb.00468-24-s0005.xlsx Tables S1 to S8.

ASM does not own the copyrights to Supplemental Material that may be linked to, or accessed through, an article. The authors have granted ASM a non-exclusive, world-wide license to publish the Supplemental Material files. Please contact the corresponding author directly for reuse.
