# Supplementary material for: Impacts of perR on oxygen sensitivity, gene expression, and murine infection in Clostridioides difficile 630∆erm
Source: J Bacteriol. 2025 Jan 23;207(2):e00468-24. doi: 10.1128/jb.00468-24 (PMC11841134; doi:10.1128/jb.00468-24)
Supplement: Figure S1 — Growth curves of (A) 630Δerm and (B) 630Δerm perRWT grown in mRCM at 0, 1, 2, and 3% O2. [file jb.00468-24-s0001.pdf]

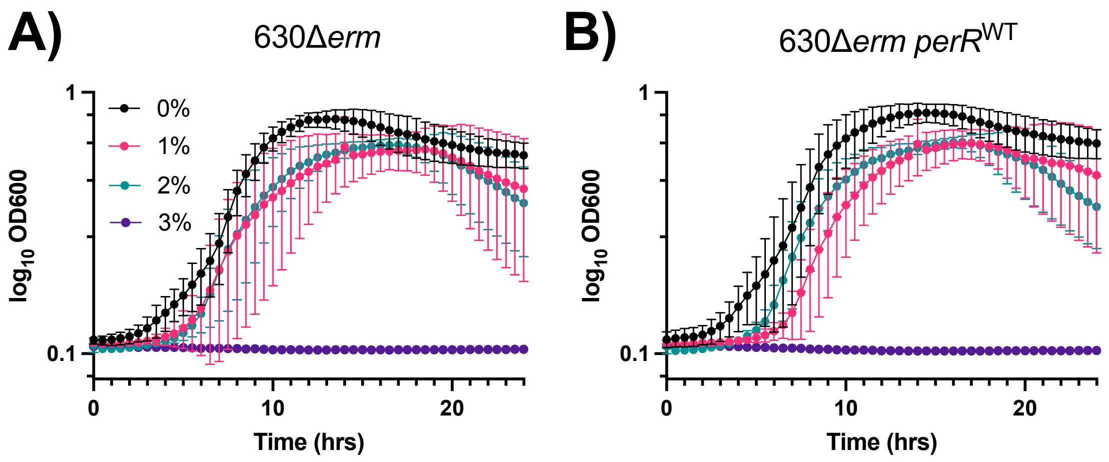

Figure S1: Growth curves of (A) 630Δ*erm* and (B) 630Δ*erm perR*^{WT} in grown in mRCM at 0, 1, 2, and 3% O_2. Data points represent the mean OD_{600} (n = 6-8 cultures per strain per condition). Error bars represent the standard deviation. Related to Figure 1.
