# Supplementary material for: Impacts of perR on oxygen sensitivity, gene expression, and murine infection in Clostridioides difficile 630∆erm
Source: J Bacteriol. 2025 Jan 23;207(2):e00468-24. doi: 10.1128/jb.00468-24 (PMC11841134; doi:10.1128/jb.00468-24)
Supplement: Figure S2 — RT-qPCR of perR transcripts from 630. [file jb.00468-24-s0002.pdf]

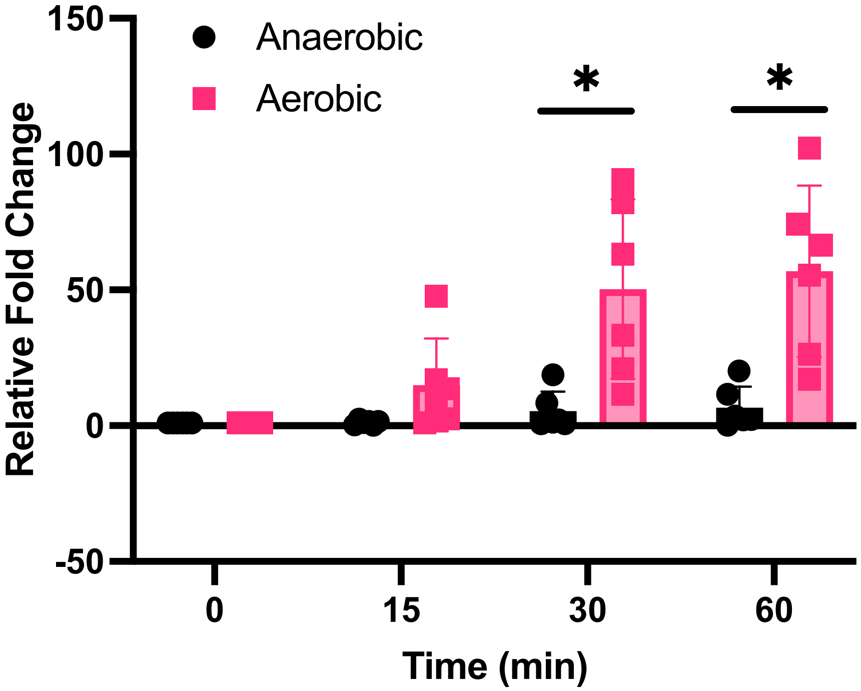

Figure S2: RT-qPCR of *perR* transcripts from 630. 630 was maintained under anaerobic conditions or exposed to ambient air for 0, 15, 30, and 60 minutes (n = 6 per strain per condition per time point). The relative fold change was determined by comparing the CT value against the average CT at 0 minutes for each condition. Statistical significance was determined by paired t-test; *, $P < 0.05$. Related to Figure 3.
