# Supplementary material for: Impacts of perR on oxygen sensitivity, gene expression, and murine infection in Clostridioides difficile 630∆erm
Source: J Bacteriol. 2025 Jan 23;207(2):e00468-24. doi: 10.1128/jb.00468-24 (PMC11841134; doi:10.1128/jb.00468-24)
Supplement: Figure S3 — Growth curves of 630, 630Δerm, and 630Δerm perRWT in the presence of clindamycin. [file jb.00468-24-s0003.pdf]

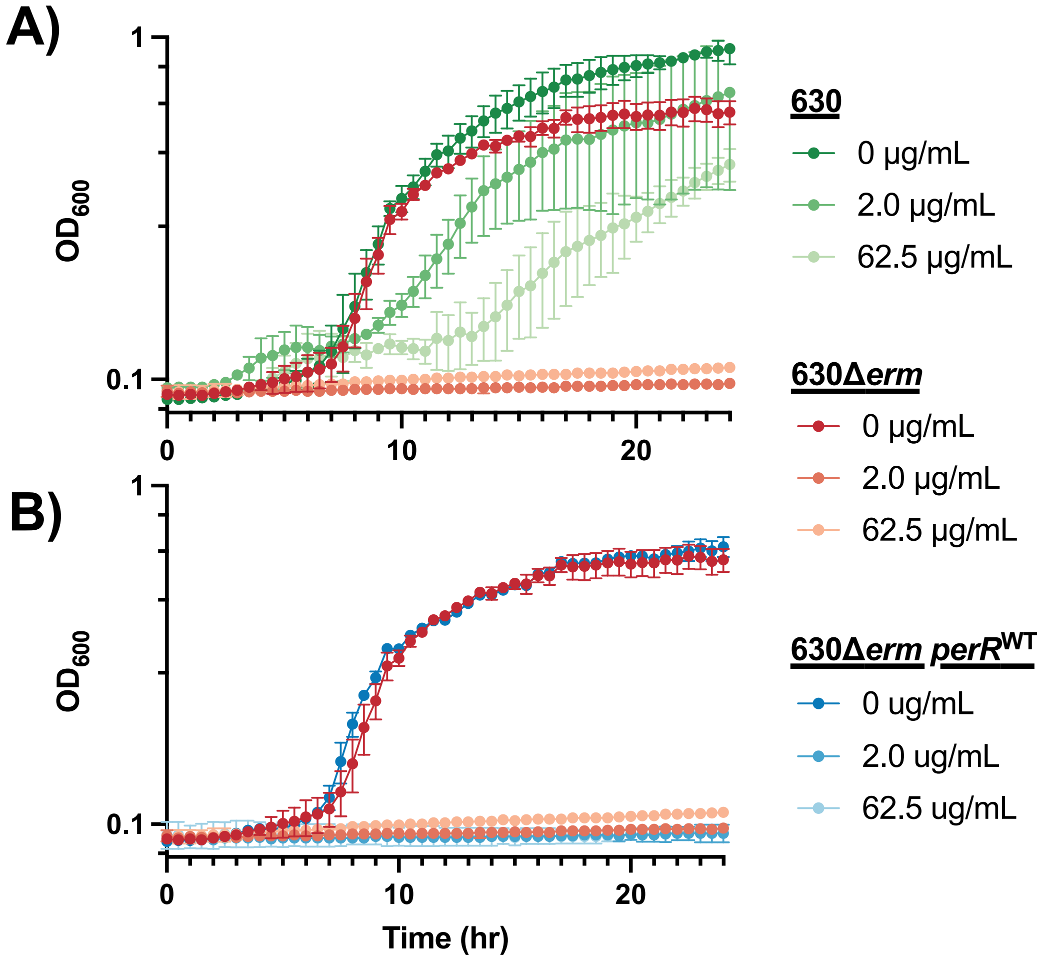

Figure S3: Growth curves of 630, 630Δ*erm,* and 630Δ*erm perR*[WT] in the presence of clindamycin. Strains were grown in mRCM containing 0, 2.0, and 62.5 µg/mL clindamycin. Data points represent the mean OD_{600} (n = 2 cultures per strain per condition) and error bars represent the standard deviation. Growth in the presence of clindamycin was compared between (A) 630 to 630Δ*erm,* and (B) 630Δ*erm* and 630Δ*erm perR*[WT]. Related to Figure 4.
