# Supplementary material for: Impacts of perR on oxygen sensitivity, gene expression, and murine infection in Clostridioides difficile 630∆erm
Source: J Bacteriol. 2025 Jan 23;207(2):e00468-24. doi: 10.1128/jb.00468-24 (PMC11841134; doi:10.1128/jb.00468-24)
Supplement: Figure S4 — HypC4III digest of perR-containing PCR amplicons. [file jb.00468-24-s0004.pdf]

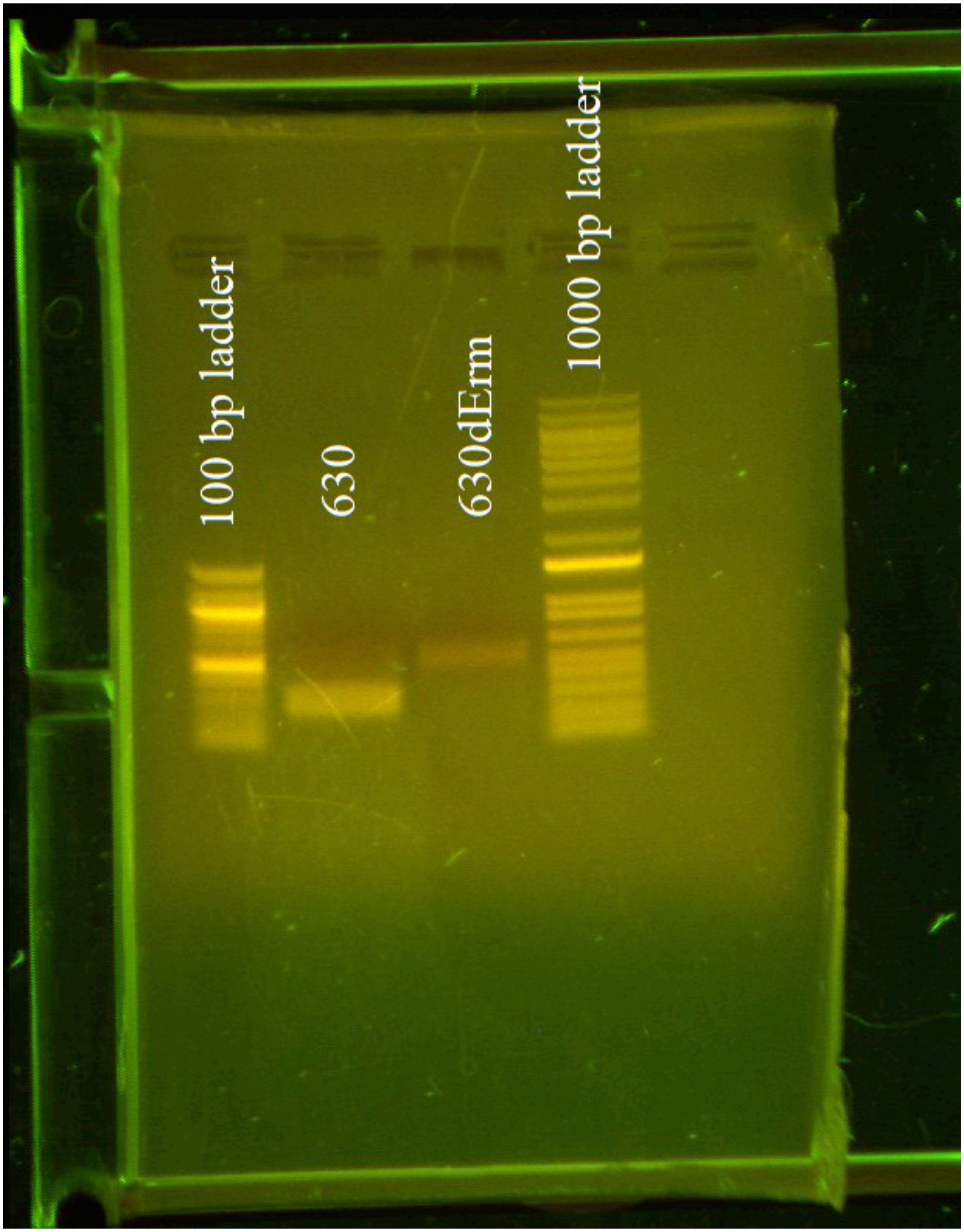

Figure S4: HypC4III digest of *perR*-containing PCR amplicons. Amplicons were generated from 630 and 630Δ*erm*. Due to the point mutation in *perR* in 630Δ*erm*, HypC4III digests the amplicon from 630Δ*erm* differently than 630. This technique was implemented to screen clones to identify 630Δ*erm perR*^{WT} candidates. Related to Figure 1.
